# Glances at the Insane in Other Lands

**Published:** 1889-01-26

**Authors:** 


### Glances at the Insane in Other Lands.

By a Medical Superintendent.

*(Continued from p. 232.)*

### IV.—FIFTY YEARS AT THE VERMONT ASYLUM.

From the State of Vermont a very interesting volume is wafted towards us. It is a neatly-bound, illustrated octavo of three hundred pages, and it presents us with a carefully-detailed, well-written history of the asylum during the last fifty years. The institution had its origin in a bequest of Mrs. Marsh, widow of Dr. Purley Marsh. We presume we ought to say "Mrs. Anna Marsh," but that is an Americanism we really cannot get used to. The lady survived till 1834, and it was then that the legacy fell into the hands of the trustees, a charter being obtained shortly afterwards. The good lady seems to have had a touch of New England humour in her composition, for we find, in her bequest for providing preachers for the town of Vernon, she remarked they were a very godless people over there.

Dr. Draper, the author of the volume, reminds us that not much was known of the treatment of the insane in the early part of this century. He tells that a council of physicians sat on a case of a prominent political man who had lost his reason, and decided to immerse the patient in water until he lost consciousness. They would then awaken him or resuscitate him to a new life. This was actually tried with the honourable gentleman, but it did not succeed. It was then discovered by the jury of experts that opium was "the proper agent for the stupefaction of the life forces." This treatment also was tried, and the patient died. In those days the bulk of the people believed that insanity was owing to supernatural causes, and this heroic treatment on the part of the physicians would have as little effect in dispelling the delusions of the mob as it would the delusions of the patient.

The Vermont Asylum was opened on the 12th day of December, 1836. In their announcement that the hospital was ready for occupation the trustees stated "that it should be borne in mind that in the first three months of insanity the chances of recovery by proper treatment are vastly greater than at any subsequent period." It is curious that fifty years later this advice has to be reiterated on both sides of the Atlantic, and too often it is absolutely ignored.

In the record for 1837 we find that 34 patients remained in the hospital. The report of the trustees says that useful labour for convalescents and all chronic cases is the best moral means that can be made use of in the treatment, and it would seem that at this early date no restriction was necessary in the use of tools, either in the garden, the wood-yard, or on the farm, and no accident took place during the year. There was only one death, and as fourteen patients were discharged, the record of the first year was most encouraging. We are specially told that neither chains nor strait-jackets were provided in the institution. One of the admissions during 1837 was a clergyman named H——. He was suffering from melancholia. He had a sister insane, and a brother had died insane. When in the asylum he wrote the following lines, which were made part of his record:—

'Twas phrenzy's hand that sadly marred
  A sister's fairest form:
My only brother's boat went down
  In just so wild a storm.
And such, I fear, will be my fate
  On life's dark raging sea;
But I must trust in Providence
  Wherever I may be.

The likening of a boat going down in a storm to the death of a brother raging mad is really very poetical. The lines strike us as being singularly like those of an old song named "The Pilot," which, if we remember correctly, used to be sung by Sir William Don.

In the record of 1838 we find the medical superintendent, Dr. Rockwell, insisting "that insanity is in all cases a corporeal disease, existing essentially in that class of material instruments or organs of the brain which are immediately and exclusively subservient to the function of the mind." Every asylum physician can corroborate the following statement, which we extract from the record of 1840: "Another mistake in regard to the insane is the premature removal of the patient from the asylum. It frequently happens that when a patient is placed at a lunatic asylum his wild and violent conduct soon gives way to that of calmness and comparative quietude, although he may be far from being restored. In the meantime he is visited by some of his friends and acquaintances, who, finding him orderly in his behaviour, sadly misjudge that he might as well be at home as in the asylum."

During this year some patients were admitted in chains, and in one case the chain had worn through the flesh to the bone of the leg. In most of the early years the proportion of recoveries was very high, being sometimes 88 per cent. of the recent cases and 28 per cent. of the chronic cases admitted.

In 1843 a small newspaper, called *The Asylum Journal*, was started, and it evidently had a fair circulation, as not fewer than two hundred exchange journals were received. It is said to be the first journal ever printed in and issued by an asylum.

In the year 1845 the character of the hospital underwent a change. Hitherto it had been conducted like a proprietary establishment, but it now became a public institution, and considerable additions were made to the buildings.

In 1848, if not earlier, the practice of allowing patients out on parole was common.

Dr. Rockwell seems to have been a man of great ability. The extracts from his reports show that his ideas were in advance of those of his contemporaries and also that he was a man of observation. In 1852 he wrote: "Most of our patients who recover cherish the kindest feeling towards those who have assisted in their recovery. Many of those who do not recover indulge the same hard feelings towards those who take care of them here, which they entertained towards their best friends before they left home." This is as true of patients now as it was then. It is the rarest thing for a recovered patient to make any complaint against the asylum; so rare, indeed, that when the complaint is made we incline to the idea that perfect recovery has not taken place.

In 1853 the asylum passed through an epidemic of small-pox, and 27 patients were attacked. In 1855 there were nearly 400 patients resident, showing to what an extent the asylum had grown in its twenty years of usefulness. In 1860 large additions were made, to keep pace with the ever-increasing admissions. Dr. Rockwell states in his report for this year that insanity is evidently increasing in the States, and he attributes it to the lax manner in which the rising generation were being brought up; to overtasking the young mind, and to the hazardous speculations in which the people were embarking. Whether insanity really increased in higher proportion than could be accounted for by the increase in the population of the States is another matter, but the above was Dr. Rockwell's opinion.

In 1862 a fire broke out in the asylum, causing a good deal of damage and the loss of four patient' lives.

In 1871, after thirty-six years' service, Dr. Rockwell resigned the superintendency, owing to ill-health. He was succeeded by his son, who accepted office for a limited time only, and in 1873 the asylum passed into the hands of its present able and accomplished superintendent, Dr. Draper. In 1879 many improvements were carried out. Dr. Draper has the following in his report, which we think well worthy of extraction: "Outside the domain of recognised insanity there is much actual mental unsoundness. The humanitarian who directs his attention to the different families of dependents upon public bounty will find little difficulty in recognising whole classes who represent the waning phases of mentality. If he visits the almshouses or eleemosynary public institutions, he is struck with the weakness and incapacity prevailing among the inmates, still more strikingly evident in the family histories he investigates, which are a mingled record of thriftlessness, want of moral sense, petty dissipations and vices—all the outcome of chronic constitutional qualities. If he visits the reformatories and prisons he will recognise a somewhat different type of mentality—still a distinctive one—which likewise is connected with a preceding line of erratic ancestry. More intellectual capacity will be found to characterise criminal psychology, but this is associated with such perversions or absence of moral sense as renders the possession of intellectual power all the more dangerous."

The fiftieth anniversary of the opening of the asylum fell on December 12th, 1886, and with this year the present volume ends. It is a little curious to notice that the asylum contained one patient who was admitted during the first year, and who had therefore been an inmate for very nearly half-a-century.

In giving us a history of the Vermont Asylum for fifty years in a readable, accurate form, and easy of reference, we believe that Dr. Draper has set an example which will be widely followed, not only in the United States, but in England. Certainly, it ought to form a precedent.

